# Gram-Negative Enterobacteria Induce Tolerogenic Maturation in Dexamethasone Conditioned Dendritic Cells

**DOI:** 10.1371/journal.pone.0052456

**Published:** 2012-12-27

**Authors:** Raquel Cabezón, Elena Ricart, Carolina España, Julián Panés, Daniel Benitez-Ribas

**Affiliations:** 1 Department of Gastroenterology, Hospital Clínic de Barcelona, IDIBAPS, Barcelona, Spain; 2 Centro de Investigación Biomédica en Red de Enfermedades Hepáticas y Digestivas (CIBERehd) and Centre Esther Koplowitz, Barcelona, Spain; Murdoch University, Australia

## Abstract

Dendritic cells have been investigated in clinical trials, predominantly with the aim of stimulating immune responses against tumours or infectious diseases. Thus far, however, no clinical studies have taken advantage of their specific immunosuppressive potential. Tolerogenic DCs may represent a new therapeutic strategy for human immune-based diseases, such as Crohn’s disease, where the perturbations of the finely tuned balance between the immune system and the microflora result in disease. In the present report, we describe the generation of tolerogenic DCs from healthy donors and Crohn’s disease patients using clinical-grade reagents in combination with dexamethasone as immunosuppressive agent and characterize their response to maturation stimuli. Interestingly, we found out that dexamethasone-conditioned DCs keep their tolerogenic properties to Gram-negative bacteria. Other findings included in this study demonstrate that the combination of dexamethasone with a specific cytokine cocktail yielded clinical-grade DCs with the following characteristics: a semi-mature phenotype, a pronounced shift towards anti-inflammatory versus inflammatory cytokine production and low T-cell stimulatory properties. Importantly, in regard to their clinical application, the tolerogenic phenotype of DCs remained stable after the elimination of dexamethasone and after a second stimulation with LPS or bacteria. All these properties make this cell product suitable to be tested in clinical trials of inflammatory conditions including Crohn’s disease.

## Introduction

Dendritic cells (DCs) represent the most potent antigen-presenting cells linking innate and adaptive immune responses. DCs express a set of receptors involved in pathogen recognition. Known as pattern-recognition receptors (PRR), they include Toll-like receptors (TLR), C-type lectins and the cytoplasmic NOD family, as well as RIG-I and MDA-5 molecules [1]. Interaction of these receptors with their specific ligands leads to DC differentiation to an activated state. Their role in the immune system is crucial, either by initiating effective immune responses or by inducing tolerance, depending on the presence or absence of danger associated molecular patterns within endocytosed particles [2].

Due to their physiological properties [3] DCs have been safely and successfully used in clinical trials aimed at stimulating an efficient immune response against tumors in humans [4], [5]. However, only one recent study has taken advantage of their specific tolerogenic properties by utilizing CD40, CD80 and CD86 antisense transfected DCs to treat diabetic patients [6]. The tolerogenic properties of immature autologous DCs have already been documented in healthy human volunteers, providing proof of principle that systemic antigen-specific T-cell tolerance can be achieved using this approach in humans [7]. However, an important concern when designing DC-based immunotherapy protocols is whether immature DCs might inadvertently receive *in vivo* maturation signals in an inflammatory microenvironment, either from pro-inflammatory cytokines and/or pathogen-derived molecules or whole microorganisms [8]. An alternative to the use of immature DCs is to generate tolerogenic DCs (tol-DCs). The addition of immunosuppressive agents, pharmacological modulation, or inhibitory cytokines during the process of DC differentiation from monocytes influences the functional properties of the resulting cells [9], [10]. Recently, a study between clinical-grade DCs compared the phenotypic characterization of human DCs using different tolerogenic agents [11]. These studies demonstrate that activation of tol-DCs might actually be a critical step in optimizing the re-stimulation and/or expansion of functional Tregs rather than in maintaining their immaturity [12], [13]. Alternative activated DCs differentially regulated naïve and memory T cells; specifically, naïve T cells were sensitized and polarized towards a low IFN-γ/high IL-10 cytokine profile, whereas memory T cells were anergized in terms of proliferation and cytokine production [14]. The studies described above were carried out using animal models or DC lines [15], [16]. However, the use of reagents that fail to fulfil GMP requirements, such as LPS, cytokines or fetal calf/bovine serum [17], makes this approach unfeasible for human trials [18]. An important obstacle to overcome in translating this method to a human setting is the need for reproducible, high-quality stable tol-DCs [19]. Furthermore, given the importance of genetic predisposition in the majority of immune mediated inflammatory disorders, it needs to be proven that tol-DCs produced from patients’ monocytes have the same tolerogenic functions as those of healthy controls.

In this study, we characterized the tolerogenic properties of monocyte-derived DCs from healthy donors and Crohn’s disease patients generated under clinical-grade conditions. In addition, we evaluated not only the stability of the tolerogenic phenotype after washing out all of the factors, but also the activation profile of those cells when exposed to different Gram-negative enterobacteria a physiologic stimuli that tol-DCs will likely encounter after administration to patients. This approach takes advantage of the complexity of the microbes that provide, at the same time, a variety of stimuli for innate receptors to elicit polarizing cytokines.

## Materials and Methods

### Generation of Human DCs and Cell Cultures

The present study was approved by the Ethics Committee at the Hospital Clinic of Barcelona. Buffy coats were obtained from Banc de Sang i Teixits and written informed consent was obtained from all blood donors. PBMC from Crohn’s disease patients were obtained with written informed consent to participate in the study. DCs were generated from the peripheral blood samples as previously reported [4]. In summary, PBMCs were allowed to adhere for 2 h at 37°C. Non-adherent cells peripheral blood lymphocytes (PBLs) were gently removed, washed, and cryopreserved. The adherent monocytes were cultured in X-VIVO 15 medium (BioWhittaker, Lonza, Belgium) supplemented with 2% AB human serum (Sigma-Aldrich, Spain), IL-4 (300 U/ml), and GM-CSF (450 U/ml) (Both from Miltenyi Biotec, Madrid, Spain) for 6 days in order to obtain immature DCs (iDCs). The maturation cocktail consisted of IL-1β, IL-6 (both at 1000 IU/ml), TNF-α (500 IU/ml) (CellGenix, Freiburg, Germany) and Prostaglandin E2 (PGE2, 10 µg/ml; Dinoprostona, Pfizer) and was added on day 6 for 24 h. Mature DCs (mDCs) were harvested and analyzed on day 7. Dexamethasone (10^−6^ M; Fortecortin, MERCK, Spain) was added on day 3. For cell stability, DCs were washed and further stimulated for 24 h with 100 ng/ml LPS (Sigma Aldrich) or 1 µg/ml of recombinant soluble CD40 ligand (Bender Medsystems, Vienna, Austria). We did not observe differences in viability and yield between iDCs, mDCs and tol-DCs generation. The protocol and reagents for tol-DC generation are fully compatible with cGMP regulations and it has been approved by Agencia Española del Medicamento y Productos Sanitarios.

Heat-killed *Escherichia coli*, *Protheus mirabillis, Klebsiella pneumoniae and Salmonella thyphimurium* were incubated at 1∶10 (DC:bacteria) ratio with DCs for 24 h. After co-incubation, supernatant was collected for cytokines determination and DCs phenotype was then analyzed.

### Flow Cytometry

To characterize and compare the phenotype of the DC populations, flow cytometry was performed. The following mAbs or appropriate isotype controls were used: anti- CD14 (eBioscience, San Diego, CA), CD80, CD83, CD86 (BD-Pharmingen), CCR7, MHC class I (W6/32 a generous gift from Dr. Ramon Vilella, Dept of Immunology Hospital Clinic de Barcelona) and FITC-labeled MHC class II (BD-Pharmingen). Primary antibodies were followed by staining with PE-labelled goat-anti-mouse (from BD Pharmingen™). Flow cytometry was performed using a FACSCalibur™ with CellQuest software (BD Biosciences) and data were analyzed using WinMDI software (version 2.9; http://facs.scripps.edu/software.html), FACSCanto II, and analyzed with BD FACSDiva 6.1™ software.

### T-cell Stimulation

For co-culture experiments, PBLs and naïve CD4^+^ T cells were isolated from healthy individuals using the CD4^+^ and naïve CD4^+^ T isolation kit (Miltenyi Biotec, Spain), according to the manufacturer’s instructions. The allo-response was tested in a mixed lymphocyte reaction; allogeneic T cells were co-cultured with DCs differently generated in a 96-well microplate. For Ag-specific T-cell responses, 1 µg/ml of tetanus toxoid (TT) (Sigma-Aldrich, Spain) or 10 ng/ml of superantigen toxic shock syndrome toxin-1 (TSST-1) (Sigma-Aldrich, Spain) loaded DCs were co-cultured with autologous T lymphocytes in a 96-round well microplate. For the proliferation assay, a tritiated thymidine (1 µCi/well, Amersham, UK) was added to the cell cultures on day six and an incorporation assay was measured after 16 h. For some experiments T cells were labelled with CFSE and plated in fixed amounts of 10^5^ cells/well. T-cell proliferation was determined by the sequential dilution of CFSE fluorescence in positive cells, as detected by flow cytometry. TT-specific cell lines were generated by adding 1 µg/ml of TT to PBMCs for one week and further cell expansion with 50 IU/ml of IL-2 for an extra week.

### Anergy Induction

For anergy induction, 1*10^6^ of highly (>98%) purified naïve CD4^+^ CD45RA^+^ T cells were co-cultured with DCs (iDCs, mDCs and tol-DCs) in a 6-well plate for 1 week (ratio 1∶10; DC:T). After extensive washing, T cells were expanded and rested in the presence of IL-2 and IL-7 for an additional week. T lymphocytes were washed and re-stimulated by co-culturing 1*10^5^ T cells with matured DCs from the original donor at 1∶20 ratio in 96-well plates. After 6 days, plates were pulsed with ^3^H-thymidine and measured as described above.

### Cytokine Production

DC supernatants were collected and frozen after 24 h of activation. IL-10, IL-12p70, IL-23 and TNF-α from the DCs supernatants and IFN-γ and IL-10 from the T-cell cultures were analyzed by ELISA according to the manufacturer’s guidelines.

### mRNA Isolation, cDNA Synthesis, and Real-time PCR

Total RNA was isolated from DCs using an RNeasy Mini Kit (Qiagen, Germany). RNA was transcribed to cDNA using a High-Capacity cDNA Archive RT kit (Applied Biosystems, USA), and was then used to perform quantitative real-time PCR in triplicate wells with a TaqMan Universal PCR Master Mix (Applied Biosystems) containing IL-10 and IL-12p35 and ß-actin (TaqMan primers and probes; Applied Biosystems). PCRs were performed using an Applied Biosystems 7500 Fast Real-Time PCR System sequence detection system. mRNA content (x) was calculated using the formula x = 2^−ΔCt^ (where ΔCt = Ct target gene-Ct housekeeping gene) were calculated for each gene and setting using ß-actin as a housekeeping gene. Fold-increase expression of target genes in mDCs or in tol-DCs was determined relative to iDCs.

### Statistical Analysis

Results are shown as the mean ± SD. To determine statistical differences between the means of two data sets, the paired or independent sample two-tailed Student *t-*tests were used. Statistically significant difference was set at *p*<0.05.

## Results

### Tolerogenic DCs Display a Semi-mature Phenotype

**Figure 1:**
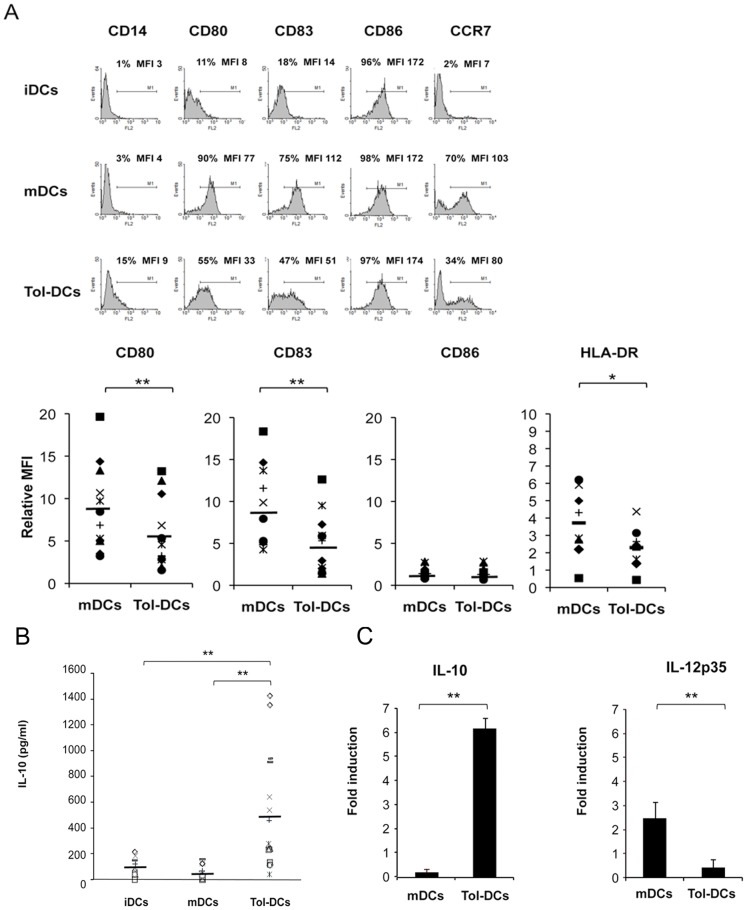
Dexamethasone modulates cytokine cocktail-induced DC maturation. (**A**) Phenotypic analysis of untreated (iDCs), cytokine-activated (mDCs) and 10^−6^ M dexamethasone cytokine-activated dendritic cells (Tol-DCs) was performed by flow cytometry. Representative histogram data set from 12 independent experiments is shown. Maturation associated molecules are depicted in the lower graph as mean fluorescent intensity of expression (MFI) of mDCs and Tol-DCs relative (fold-change expression) to iDCs. (**B**) IL-10 and IL-12p70 were measured in supernatants harvested from DCs. Concentration of IL-10 (in pg/ml) is shown (n = 15). In none of the conditions analyzed were IL-12p70 or IL-23 produced (lowest detection limit 7.6 pg/ml). (**C**) Transcripts levels of IL-10 and IL-12p35 were determined by real-time PCR using β-actin as the endogenous reference gene. Data represent fold-change induction relative to iDCs (n = 3). Student’s *t*-test: *p<0.05, **p<0.001.

The presence of dexamethasone during DC diferentiation partially impaired the upregulation of co-stimulatory molecules such as CD80 (38% reduction, p<0.001), the maturation marker CD83 (40% reduction, p<0.001), and the HLA-DR (39% reduction, p<0.05) compared with fully mDCs (**Figure 1A**). CD86 was highly expressed on iDCs and we did not observe any significant changes in the expression of CD86 upon activation in tol-DCs compared to mDCs. Consistently, similar phenotypic results were obtained by stimulation of dexamethasone-treated DCs with TLR ligands, such as LPS (data not shown), as elsewhere described [20], [21], [11]. The maturation of DCs resulted in a tightly regulated production of pro- and anti-inflammatory cytokines, depending on the type of stimuli. In accordance with the tolerogenic phenotype shown in **Figure 1A**, tol-DC cytokine secretion resulted in significantly higher production of the anti-inflammatory cytokine IL-10 (mean = 510±453 pg/ml) compared with either iDCs (68±69 pg/ml, p<0.001) or mDCs (51±59 pg/ml, p<0.001) (**Figure 1B**). The inflammatory cytokines IL-12p70 and IL-23 remained undetectable in the supernatants of either tol-DCs or mDCs, which is coherent with the absence to TLR-L on the maturation cocktail [22], [23]. In order to confirm these results, we analyzed the transcripts of these cytokines by real-time PCR. mRNA levels for the pro-inflammatory cytokine IL-12p35 were significantly reduced in tol-DCs compared to mDCs (**Figure 1C**), whereas the RNA levels of IL-10 exhibited a significant six-fold increase in tol-DCs compared with mDCs, thus corroborating our results at the protein level.

### Tolerogenic DCs Show Reduced T-cell Stimulatory Capacity

**Figure 2:**
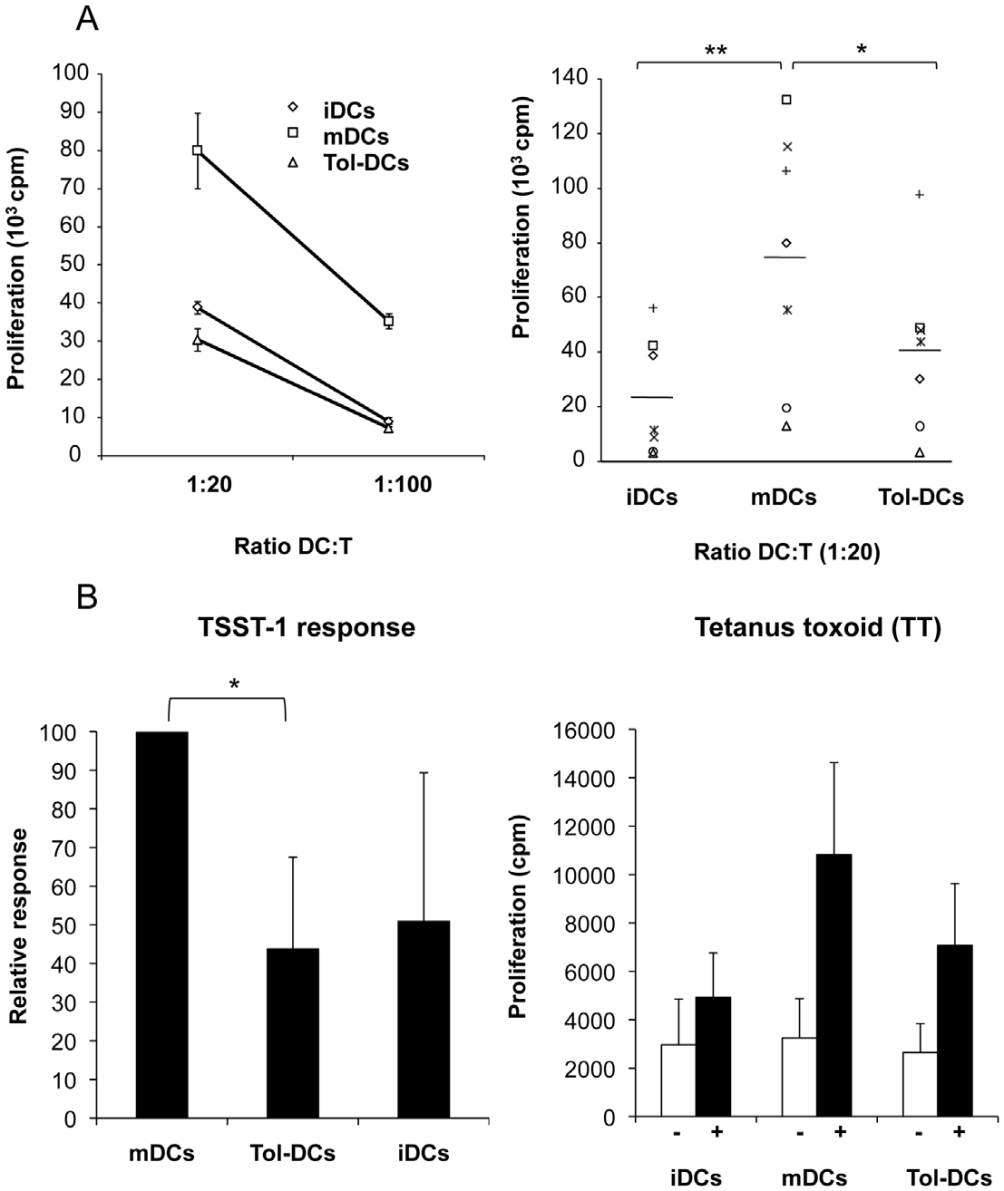
Tol-DCs have a reduced capacity to stimulate T lymphocytes. (**A**) DCs were cultured with allogeneic PBL at different ratio (1∶20 or 1∶100) for seven days. Upper-left panel data represent the mean ± SD of a representative experiment carried out in triplicate of the seven (upper-right graph) that were independently performed. (**B**) Antigen-specific T-cell responses. CD4^+^ T cells we cultured with autologous DCs pre-loaded with the superantigen TSST-1 (left graph) or with tetanus toxoid (+ presence and – absence of TT) at a 1∶20 ratio for seven days. T-cell proliferation was determined in triplicate by ^3^H thymidine incorporation. Data represent the mean ± SD of n = 3 independently performed experiments. Student’s *t*-test: *p<0.05, **p<0.001.

To determine the functional properties of clinical-grade tol-DCs, we analyzed their T-cell stimulatory capacity. Tol-DCs induced a lower proliferative allo-response (mean cpm = 40.879, p<0.05) compared to mDCs (cpm = 74.651), whereas the response to iDCs was also low (mean cpm = 23.634, p<0.001 vs mDCs) as expected, **Figure 2A**. We also investigated the capacity of tol-DCs to present exogenous antigen to autologous T cells. As depicted in **Figure 2B**, tol-DCs exhibited a reduced antigen-presenting capacity to autologous T cells compared with control DCs, when the latter were loaded with either the superantigen toxic shock syndrome toxin-1 (TSST-1) or tetanus toxoid (TT). Thus, tol-DCs were poorer stimulators of allo- or antigen-specific T-lymphocyte responses (in allogeneic and autologous settings) than mDCs.

### Tolerogenic DCs Generate Antigen-specific Anergic T cells

**Figure 3:**
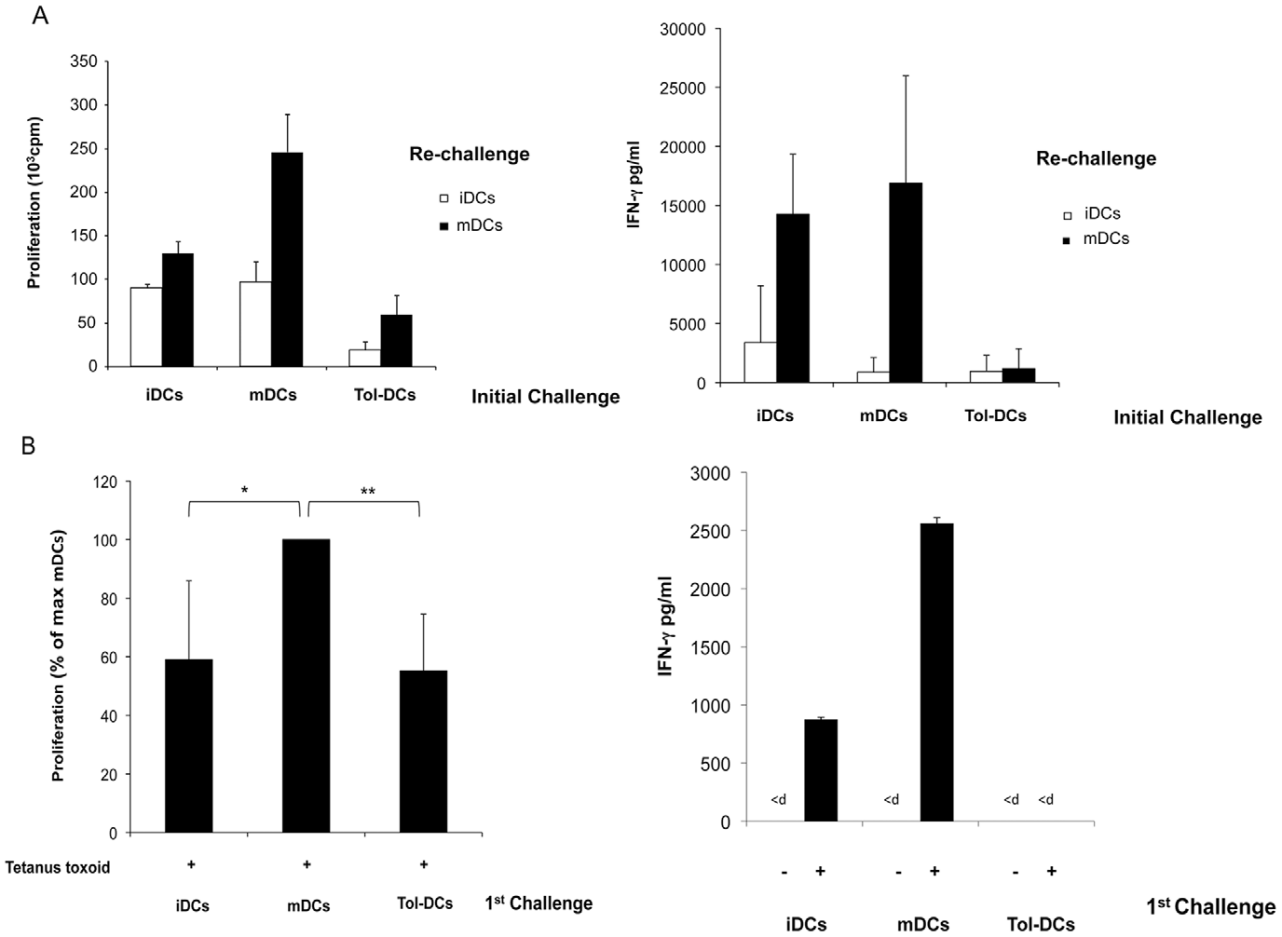
Tol-DCs induce anergic T cells. (**A**) Naïve CD4^+^ CD45RA^++^ T cells were primarily primed with allogeneic iDCs, mDCs or tol-DCs for 7 days. After 5 days, anergy induction was examined by re-stimulation of primed CD4^+^ T cells with iDCs or mDCs from the original donor. (**B**) TT-specific CD4^+^ T cells were primed with TT-loaded autologous iDCs, mDCs or tol-DCs for 6 days (initial challenge). After *in vitro* expansion with TT loaded-DCs anergy induction was examined by re-stimulation of TT-specific CD4^+^ T cells with mDCs loaded (+) with TT at a 1∶20 ratio. Data represent the mean ± SD of n = 5 experiments that were independently performed. Proliferation was normalized relative to mDCs loaded with TT (100%) for each independent experiment. Cytokines were determined in the supernatant of cell cultures by ELISA (<d; below detection limit; IFN-γ data represent mean ± SD of n = 3).

To evaluate the ability of tol-DCs to induce CD4^+^ T-cell hypo-responsiveness, allogeneic highly purified CD4^+^ naïve T cells (purity 98% CD4^+^CD45RA^+^) were initially primed for 14 days during the first round with iDCs, mDCs or tol-DCs (initial challenge) and then were re-stimulated (re-challenged) with iDCs or fully competent mDCs from the original donor. T cells exposed to tol-DCs exhibited a reduced capacity to proliferate as well as reduced IFN-ÿ secretion when re-challenged with fully competent mDCs. In contrast, T cells exposed to control DCs proliferated and secreted IFN-γ to a high degree (**Figure 3A**). To confirm the capacity of tol-DCs to mitigate effector T cells, tetanus toxoid (TT)-specific T cell lines were re-stimulated with TT loaded or control (non-loaded) mDCs. Whereas T cells primarily exposed to mDCs vigorously responded to TT, as measured by T-cell proliferation and IFN-γ production (**Figure 3B**), those exposed to tol-DCs showed a significantly reduced proliferation and an absolute inability to induce IFN-γ during a secondary response to TT-loaded DCs.

### Tolerogenic DCs are Stable and Resistant to Further Stimulation

**Figure 4:**
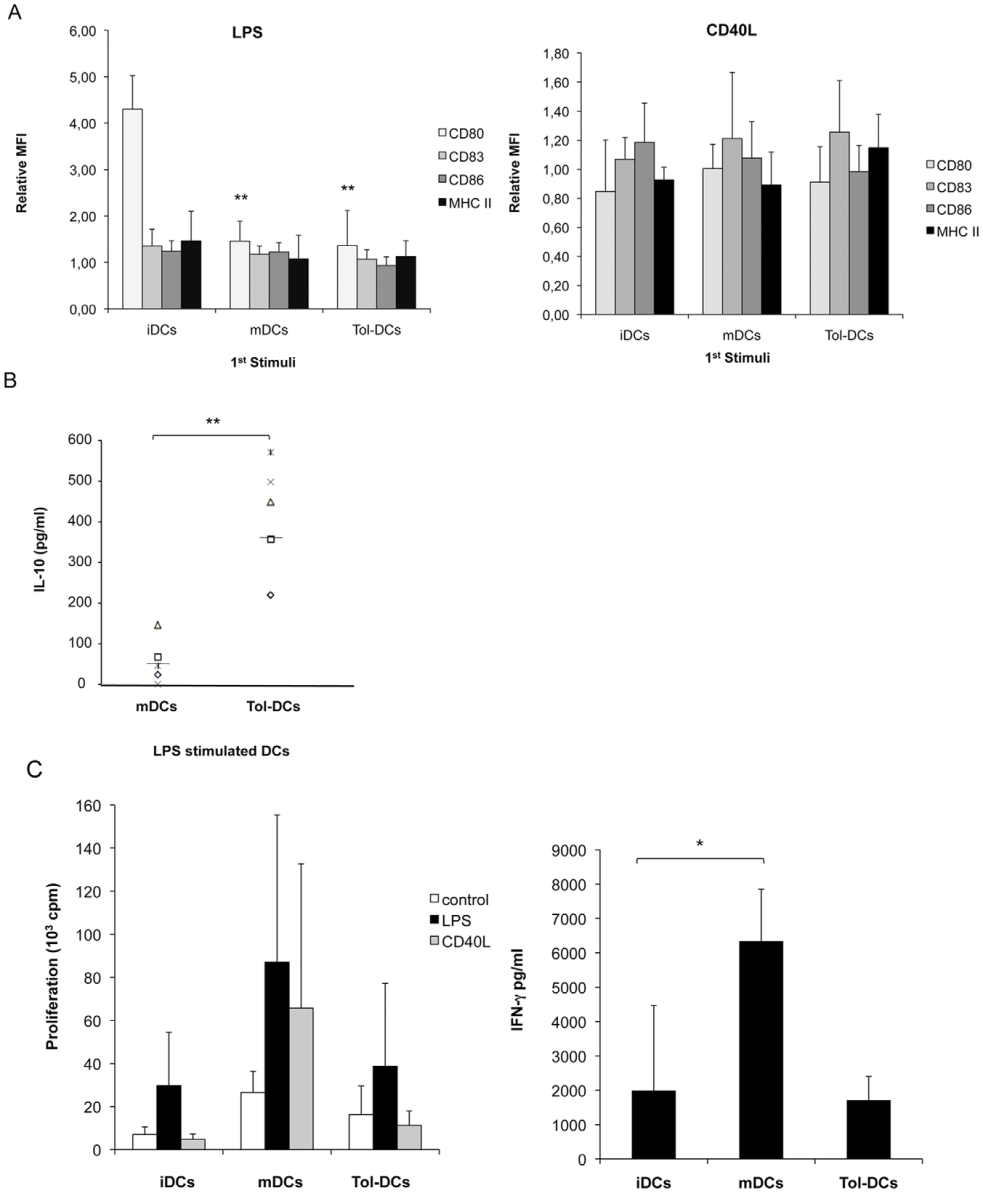
Tol-DCs possess a stable phenotype. DCs were carefully washed to eliminate cytokines and dexamethasone, and viable DCs were further re-challenged with 100 ng/ml of LPS or 1 µg/ml of soluble CD40L as second stimuli. After 24 h, the phenotype (**A**) was analyzed by flow cytometry. Data represent relative MFI increase induced by LPS (n = 6) or CD40L (n = 4) compared to unstimulated iDCs, mDCs or tol-DCs as control. (**B**) IL-10 concentration is shown in pg/ml. IL-12p70 and IL-23 were not detected (detection limit = 7.8 pg/ml). Student’s *t*-test: *p<0.05, **p<0.001. (**C**) Tol-DCs do not recover the ability to stimulate T cells after re-challenge. T-cell proliferation was determined in triplicate by ^3^H-thymidine incorporation. IFN-γ and IL-10 production in the supernatant was analyzed.

To address the stability of tol-DCs, dexamethasone and cytokines were carefully washed away and the DCs were re-stimulated with secondary maturation stimulus. Tol-DCs were refractory to further stimulation with LPS (**Figure 4A**, data from **n = 6** independent experiments) and CD40L (**n = 4**), maintaining a stable semi-mature phenotype. Interestingly, tol-DCs retained their ability to further produce high levels of IL-10, but failed to generate IL-12 or IL-23 following stimulation with LPS (**Figure 4B**) data not included for negative IL-12 and IL-23), we did not detect any cytokine after CD40L stimulation. Furthermore, tol-DCs re-challenged with LPS or CD40L were unable to induce a proliferative T-cell response (**Figure 4C**). In addition, the lower levels of IFN-γ cytokine secretion by T cells stimulated with LPS-treated tol-DCs compared with mDCs (mean 6332±1514 vs 1700±700 pg/ml p = 0.07) suggest inhibition of the Th1-type response (**Figure 4C**).

### Tolerogenic Response of Dexamethasone-conditioned DCs to Gram-negative Bacteria

**Figure 5:**
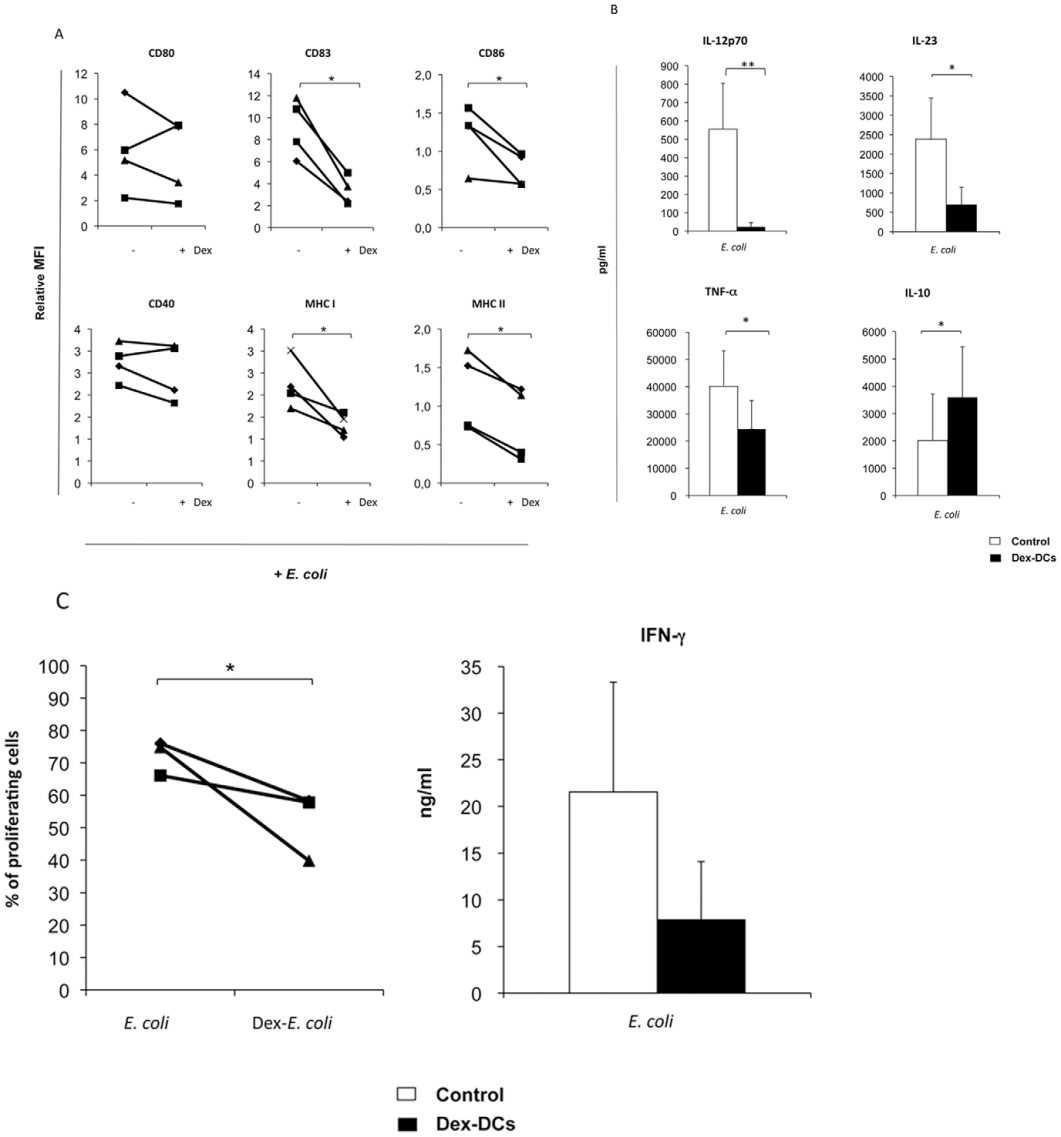
Gram-negative bacteria do not break the tolerogenic properties of dexamethasone-DCs. Heat-killed bacteria were added at ratio 1∶10 for 48 h to mo-DCs treated with dexamethasone or untreated as a positive control. **A**. Phenotypic analysis revealed statistically significant reduction of CD83, CD86, and MHC I and class II expression. Maturation associated molecules are depicted as mean fluorescent intensity of expression (MFI) of *E. coli* stimulated-DCs relative (fold-change expression) to control DCs without *E. coli*. (**B**) Cytokines produced by *E. coli-*stimulated DCs. Reduction of IL-12p70 (95.9%; p<0.05), IL-23 (70.5%; p<0.05) and TNF-α (40%; p<0.05) and elevation of IL-10 (78% increase; p<0.05) in Gram-negative treated DCs. (**C**) Gram-negative stimulated DCs were cultured after being carefully washed with allogenic PBLs (ratio 1∶20) for 7 days. The % of proliferating cells was measured by CFSE dilution using flow cytometry. Significant allo-response inhibition of *E. coli* dex-DC (inhibition 28%; p<0.05) compared to control DCs. IFN-γ secretion was analyzed in the supernatant by standard ELISA. Results represent the mean and standard deviation of three independent donors. Student’s *t*-test: *p<0.05, **p<0.001.

Whole microorganisms contain multiple PAMPs capable of stimulating DCs by different pathways. This capacity exemplifies a more physiological setting, versus the use of restricted TLR agonists or exogenous recombinant cytokines. DCs were incubated with Gram-negative heat-inactivated *Escherichia coli (E. coli)*. Interestingly, the presence of dexamethasone during DCs differentiation profoundly influenced cell maturation, exhibiting strong inhibitory effect on their phenotype (**Figure 5A**) with significant reduction in CD83, CD86 and MHC class I and II expression, when compared with DCs without *E. coli*. Importantly, it caused a robust inhibition of pro-inflammatory cytokines (IL-12p70, IL-23 and TNF-α), increased IL-10 secretion (**Figure 5B**), and modified the immune response of T lymphocytes (**Figure 5C**) inhibiting T cell proliferation and Th1 induction. The production of IFN-γ by T cells was inhibited (mean 21550±11782 pg/ml vs 7869±6198 pg/ml; p = 0.07) when DCs were conditioned with dexamethasone previously to *E. coli* stimulation. We did not detect any IL-10 in the supernatant of activated T cells.

### Tolerogenic DCs are Stable and Resistant to Further Gram-negative Bacteria

**Figure 6:**
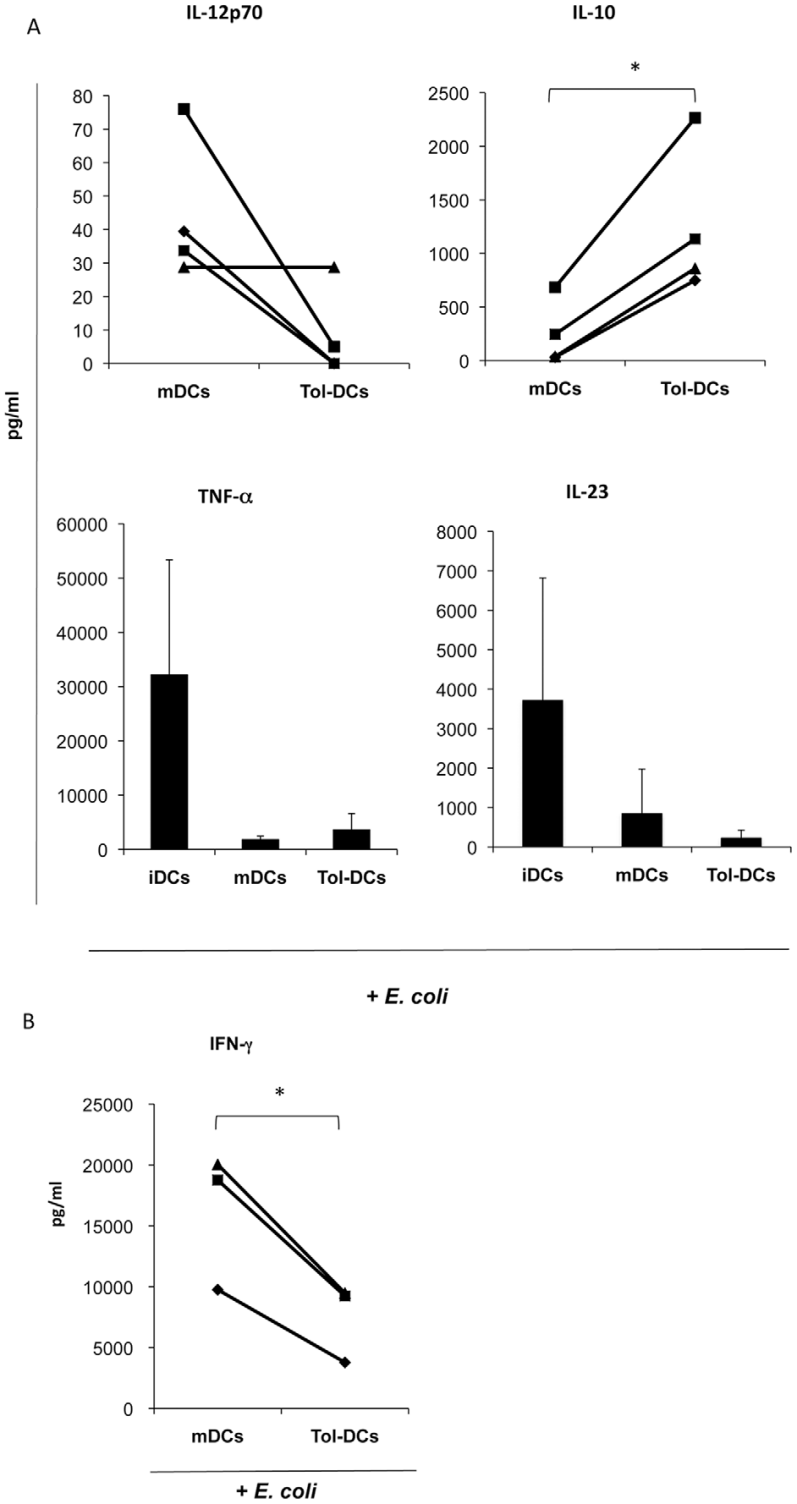
Gram negative *E. coli* induces tolerogenic activation on Tol-DCs. DCs were carefully washed to eliminate cytokines and dexamethasone at day 7, and viable DCs were further re-challenged with *E. coli* (ratio 1∶10) without cytokines or dexamethasone. (**A**) Tol-DCs (dex matured-DCs) produced significant higher levels of IL-10 whereas levels of pro-inflammatory cytokines were very low compared with mDCs or iDCs in response to *E. coli* (n = 4, from each donor, iDCs, mDCs and tol-DCs were generated in parallel). (**B**) The production of IFN-γ was evaluated in the supernatant of allogenic T cells cultured for 7 days with *E. coli* stimulated mDCs or tol-DCs. IFN-γ production was significantly (p = 0.024) reduced in T cells stimulated with tol-DCs plus *E. coli*. IL-10 was not detected in any condition (data not included). Student’s *t*-test: *p<0.05, **p<0.001.

**Figure 7:**
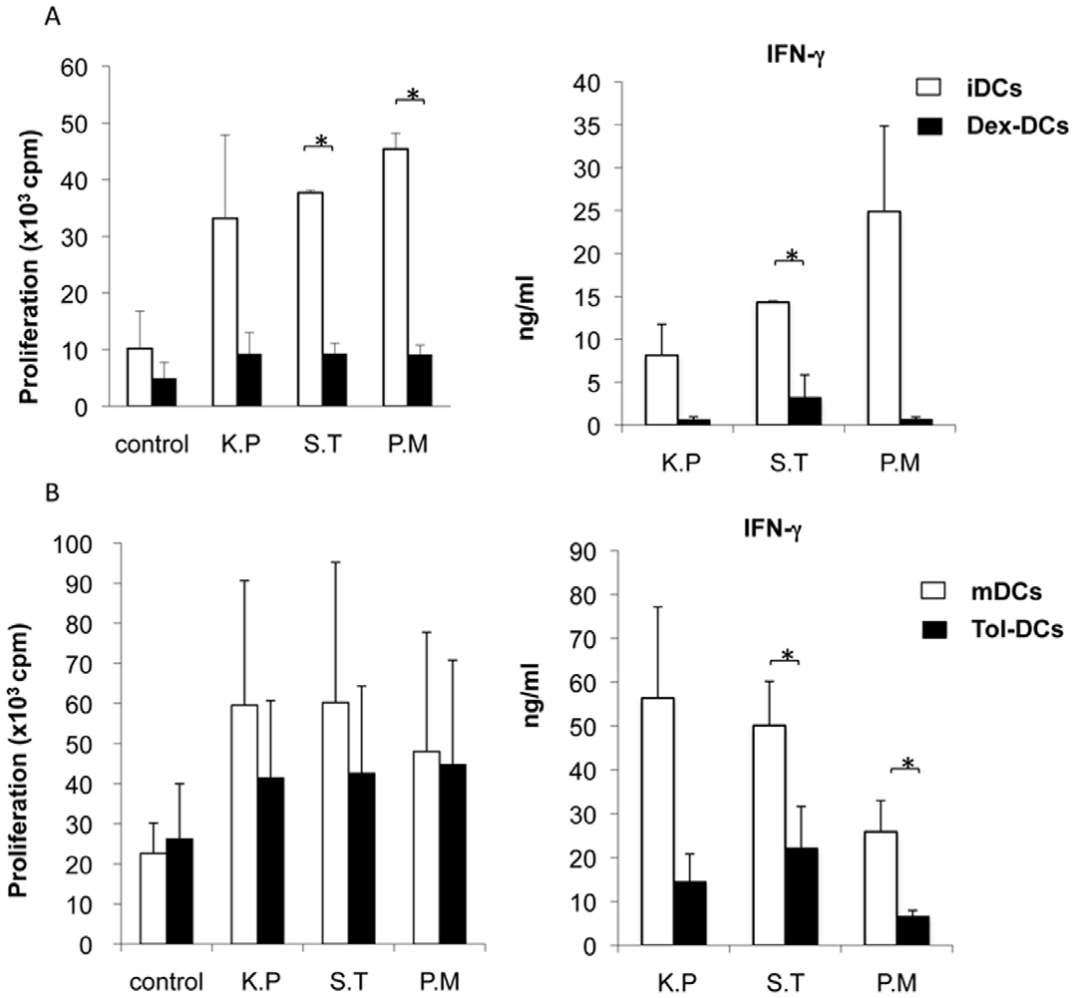
Tol-DCs interaction with Gram-negative enterobacteria inhibits Th1 response. Tol-DCs were treated as described in figure 5 and 6. Proliferative response and IFN-γ production induced by Gram-negative enterobacteria (*P. mirabillis, K. pneumoniae and S. thyphimurium)* stimulation of dex-DCs (**A**) and tol-DCs (dex matured-DCs) (**B**) were evaluated in allogeneic T cell culture. IFN-γ production was reduced in T cells stimulated with tol-DCs plus Gram-negative enterobacteria. IL-10 was not detected. Data represent mean ± SD of four independent experiments. Student’s *t*-test: *p<0.05.

To address the stability of tol-DCs, dexamethasone and maturation cytokine cocktail were carefully washed away as described above and DCs were incubated with *E. coli* for further 24 h without dexamethasone or other factors present in the culture. Tol-DCs were refractory to further stimulation with Gram-negative bacteria. Interestingly, tol-DCs produced significantly higher levels of IL-10 in response to *E. coli* than mDCs (mean 1252±694 vs 249±306 pg/ml; p = 0.01) even after DC maturation with a cytokine cocktail, whereas the levels of pro-inflammatory cytokines were hardly detected (**Figure 6A**). Furthermore, when we evaluated the capacity of DCs to generate Th1 response we observed that tol-DCs induced significant lower IFN-γ levels compared to mDCs (**Figure 6B**). The results obtained with *E. coli* were further confirmed and strengthened when different Gram-negative enterobacteria. *P. mirabillis, K. pneumoniae and S. thyphimurium* were incubated with dexamethasone-conditioned DCs (**Figure 7A**) or with tol-DCs (dex-DCs plus maturation cocktail) (**Figure 7B**) after washing out the immunosuppressive agent and cytokines. Although, mDCs and tol-DCs stimulated with bacteria provoked a comparable T cell proliferative response, the IFN-γ secretion was significantly reduced in both culture conditions (no IL-10 was detected in any condition) (**Figure 7**). These results show the incapacity of dex-DCs or tol-DCs to generate Th1 response measured by IFN-γ production revealing the stability of the tolerogenic properties, even after strong and activation induced by Gram-negative bacteria.

### DCs from Crohn’s Disease Patients can be also Educated towards a Tolerogenic Phenotype

**Figure 8:**
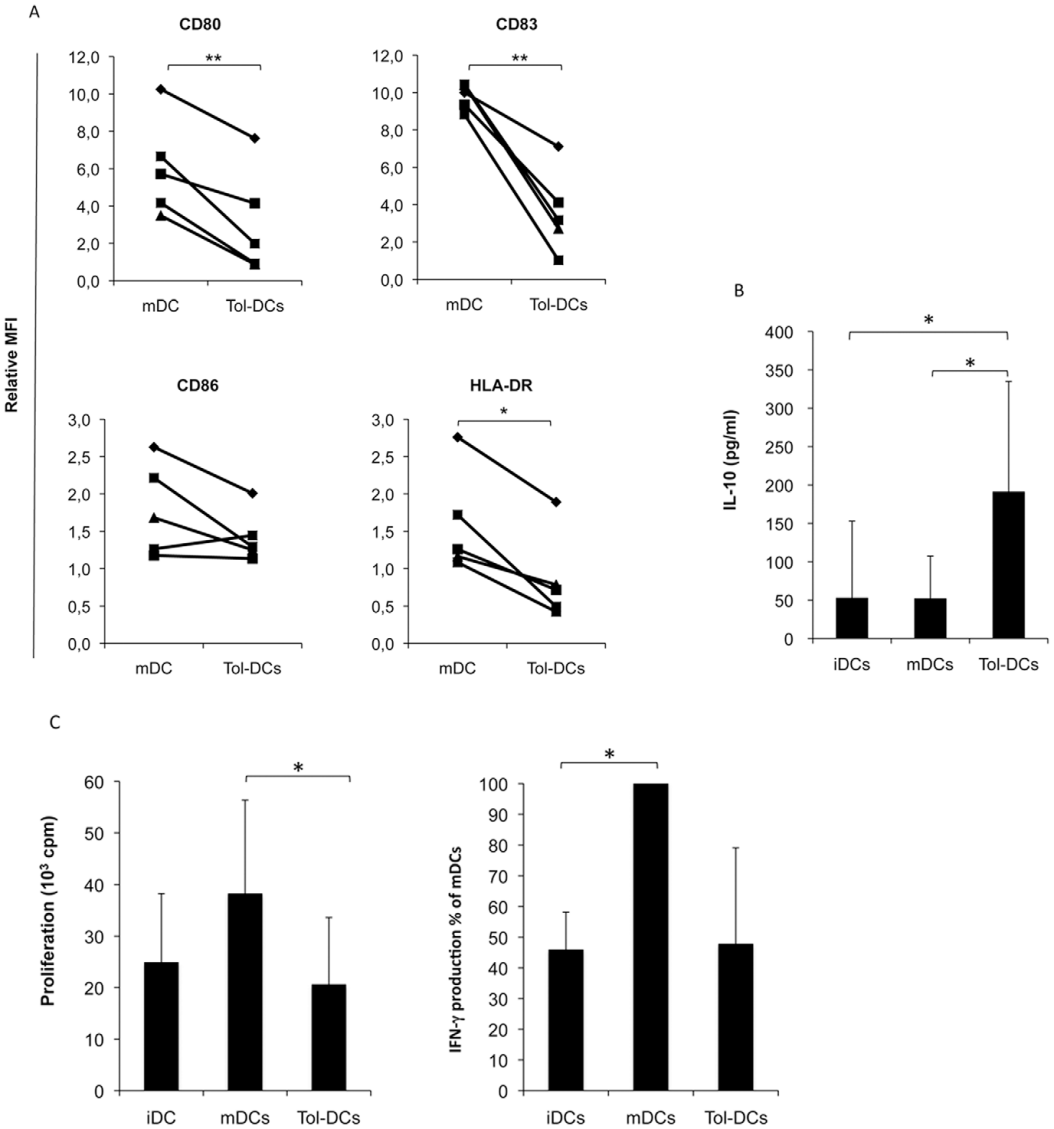
Crohn’s disease patients’ DCs are educated towards tolerogenic phenotype. (**A**) Maturation associated molecules upregulation in DCs from Crohn’s disease patients are depicted as mean fluorescent intensity of expression (MFI) in mDCs and tol-DCs relative to iDCs (fold-change expression). (**B**) IL-10 was measured in supernatants harvested from DCs. Concentration of IL-10 (in pg/ml) is shown as mean ± SD (n = 6). (**C**) Proliferative response and IFN-γ production induced by tol-DCs from patients were evaluated in allogeneic T cell culture. Both, proliferation and IFN-γ production were reduced in T cells stimulated with tol-DCs compared to mDCs (data represent mean± SD (n = 4)). IFN-γ production was normalized relative to mDCs (100%) for each independent experiment (n = 3). Student’s *t*-test: *p<0.05.

In order to validate the tol-DCs generation in the context of an inflammatory disease, DCs from Crohn’s disease patients were generated and analysed. As depicted in **figure 8A**, tol-DCs generated from Crohn’s disease patients showed a statistically significant impairment in the upregulation of CD80, CD83 and HLA-DR compared to iDCs, with no CD86 modification. Interestingly, the levels of IL-10 were significantly increased in the supernatants of tol-DCs of Crohn’s disease patients compared to mDCs and iDCs (**figure 8B**) and did not produce pro-inflammatory cytokines like IL-12 or IL-23 (data not included). Furthermore, T cells exposed to tol-DCs from Crohn’s disease patients exhibited a significantly reduced capacity to proliferate (mean cpm = 20561±13058 vs 38181±18177; p = 0.037) compared to mDCs, as well as reduced IFN-γ secretion when co-cultured with fully competent mDCs (**figure 8C**). These results show the ability to generate tol-DCs in patients with Crohn’s disease.

## Discussion

The generation of reproducible and stable clinical-grade tolerogenic DCs is a critical step towards developing therapeutic trials for the treatment of human disorders such as allergies, autoimmune diseases, chronic inflammation, and transplant rejection [19] [24]. The addition of immunosuppressive agents, pharmacological modulation, or inhibitory cytokines when DCs are being generated from monocytes influences the functional properties of the resulting DCs [9], [10]. Several agents, including glucocorticoids [25] such as dexamethasone [26], [27], mycophenolic acid [28], vitamin D3 (1α,25-dyhydroxyvitamin D_3_) [29], retinoic acid [30], the combination of dexamethasone and vitamin D3 [31], or IL-10 [32] have been used to render DCs resistant to maturation [33].

Tolerogenic DCs have been shown to induce T-cell anergy [34], suppress effector T cells, and promote the generation of regulatory T cells (Tregs) [14], [35]. Interestingly, some studies [14] have reported that the maturation of dex-conditioned DCs with LPS potentiates the tolerogenic phenotype of DCs.

We performed a detailed phenotype analysis in order to compare iDCs and fully mature DCs with tol-DCs from healthy donors and patients with Crohn’s disease and address the stability of tol-DCs. DCs conditioned with dexamethasone displayed a semi-mature phenotype, which is consistent with the tolerogenic DC phenotypes described elsewhere [36]. We also observed an alteration in the DC maturation process; characterized by low-intermediate CD80, CD83, CCR7, MHC class I and MHC class II expression. The high levels of CD86 on DCs can be explained by the presence either of human serum or steroids in the culture [37]. Indeed, dexamethasone has been shown to increase CD86 expression through GILZ (glucocorticoid-induced leucine zipper) induction [38]. Furthermore, interactions involving CD80/86 are needed in order to expand Tregs, as was revealed when Treg expansion was inhibited via the use of CD86-blocking antibodies [39]. CCR7 mediates the migration of peripheral DCs to lymph nodes [40]. Although CCR7 expression is induced on DCs by PGE2 [41], we were unable to detect CCR7 expression in tol-DCs by increasing PGE2 concentration (unpublished results). Our data clearly demonstrate that a phenotypic description alone without functional studies appears insufficient for ascertaining the nature of tol-DCs. Comparisons between different tolerogenic agents have revealed the differences among these so-called tol-DCs [11], [33]. The cytokine balance determines the type of T-cell effector response when DC-T cell interaction occurs. Pro-inflammatory cytokines like IL-12p70 and IL-23 were absent in tol-DCs at both the protein and mRNA transcripts levels. Interestingly, levels of IL-10 in response to maturation stimuli, which is one of the most important anti-inflammatory cytokines having powerful tolerogenic properties, were significantly higher in tol-DCs compared with mDCs. The balance between IL-12/IL-10 might be crucial both for the induction of tolerance and for Th1 inhibition.

Tol-DCs exhibited a low stimulatory capacity in an allogeneic-mixed leucocyte reaction, as well as skewed T-cell polarization toward an anti-inflammatory phenotype. Importantly, this immunosuppressive function was also observed in autologous settings when superantigen TSST-1 or TT antigens were used as recall antigens. DCs can be manipulated to induce T-cell anergy and regulatory T-cell activity depending on the maturation level and the interaction with naïve CD4^+^CD45RA^+^ or memory T cells. The induction of anergy on naïve T cells could represent another mechanism of tolerance induction. In our study, we demonstrate that naïve T cells expanded with tol-DCs were unable to proliferate, even after further stimulation with fully mature DCs from the same donor. Interestingly, we observed the same pattern of inhibition when TT was used as specific antigen. While TT induces strong IFN-γ secretion following interaction with mDCs [42], in our study tol-DCs completely inhibited such Th1 polarization. Increasing evidence suggests that mature DCs that lack the ability to deliver signal 3 preferentially promote the differentiation of CD4^+^ T cells into IL-10 producing T cells (reviewed by Joffre O et al. [22]). Interestingly, our results reveal that tol-DCs have the capacity to tolerize memory T cells, which are generally viewed as very difficult cell type to tolerize. However, we failed to generate *de novo* Treg (Foxp3 positive) from purified naïve CD4^+^ T lymphocyte when cultured with tol-DCs.

An important concern to be considered when designing DC-based immunotherapy protocols is their stability. In this regard, it is important to point out that tol-DCs maintained their tolerogenic properties (particularly relevant for IL-10 production) once the immunosuppressive agent was removed from the culture and the DCs were further stimulated with LPS or CD40L.

It is important to stress that the tolerogenic effects of dexamethasone were evident after adding whole microorganisms (Gram-negative enterobacteria), taking into account the presence of multiple PAMPs capable of stimulating DCs by various pathways [43], [44]. Interestingly, it has been recently described how glucocorticoids alter DC maturation in response to TLR7 or TLR8 through a mechanism involving GR transcriptional activity [45]. These results indicate that the response to commensal bacteria is directly related to any pre-conditioning DCs receive, underscoring the importance of the interaction between DCs and their surrounding environment [46]. Although pre-conditioning might entail some risk of infection in treated patients, it may also constitute a critical component in the treatment of immune-mediated inflammatory disorders, particularly of those in which an inappropriate response to commensal bacteria is believed to play a role, such as inflammatory bowel diseases. The clinical relevance of such interaction between enterobacteria with clinical-grade tol-DCs would take place in the inflamed lamina propria of IBD patients in the context of a cellular-based therapy. Importantly, we confirm for the first time that this protocol could be used for the production of tol-DCs from Crohn’s disease patients, in line with studies in other immune-based diseases like rheumatoid arthritis [47] or multiple sclerosis [48]. This is a key aspect for considering this form of cell therapy in Crohn’s disease, because it might have occurred that genetic variants conferring susceptibility for Crohn’s disease might alter the biology of DCs.

In conclusion, we herein report that DCs generated by the addition of dexamethasone in combination with a cocktail of pro-inflammatory cytokines yield clinical-grade DCs with tolerogenic properties. Tol-DCs remain stable after Gram-negative bacteria interaction. These properties may serve as the basis for modulating abnormal immune responses and for developing effective strategies for the treatment of immune-mediated diseases.
